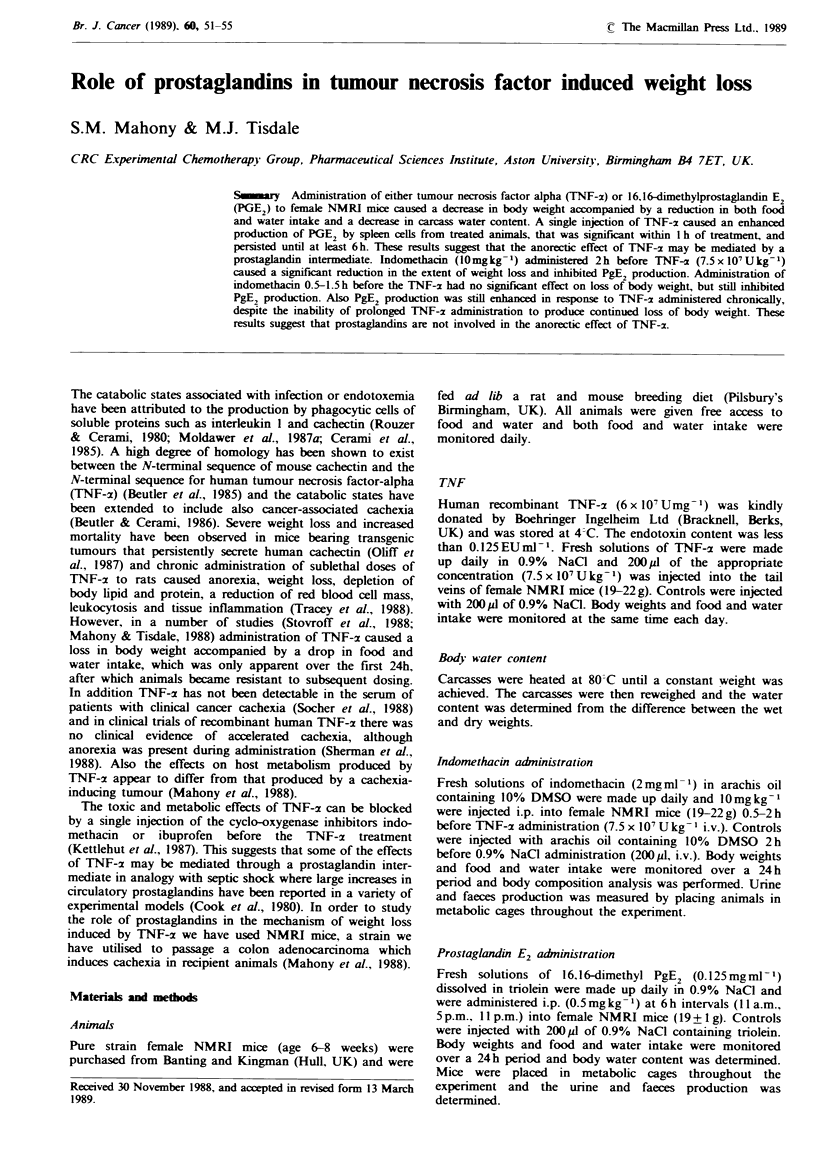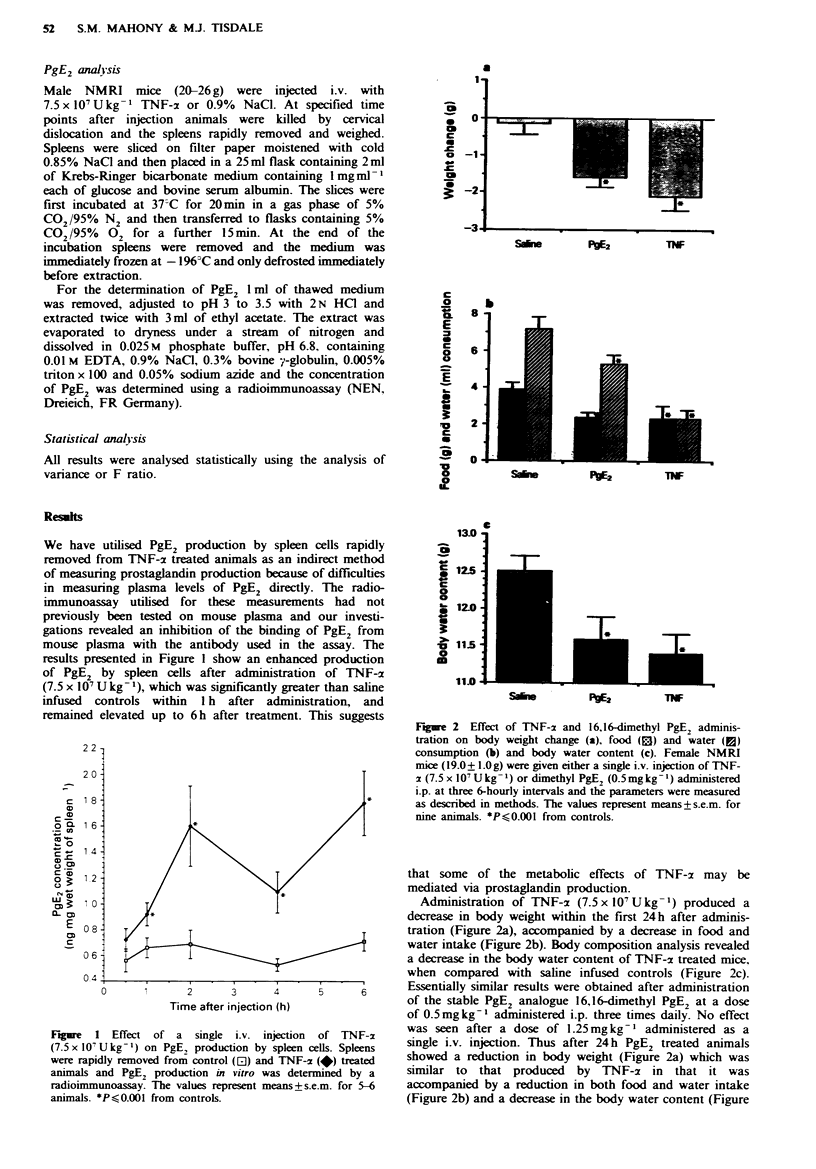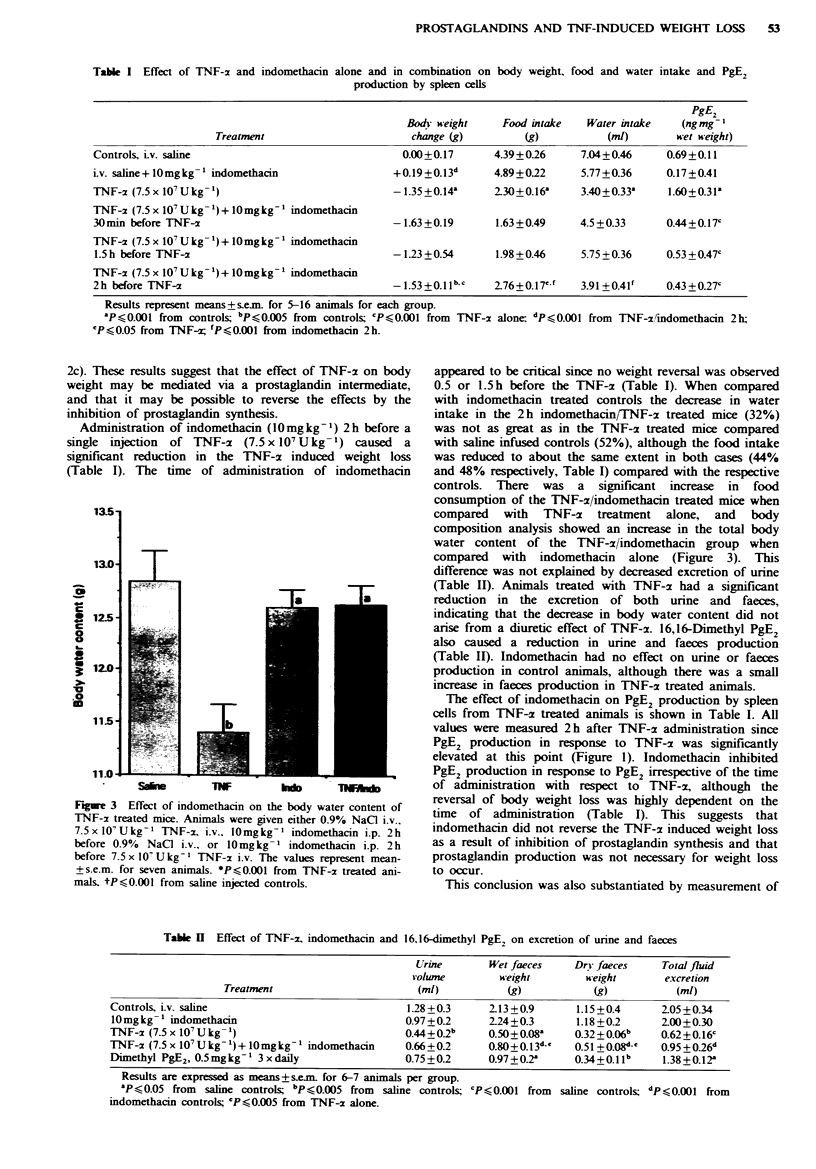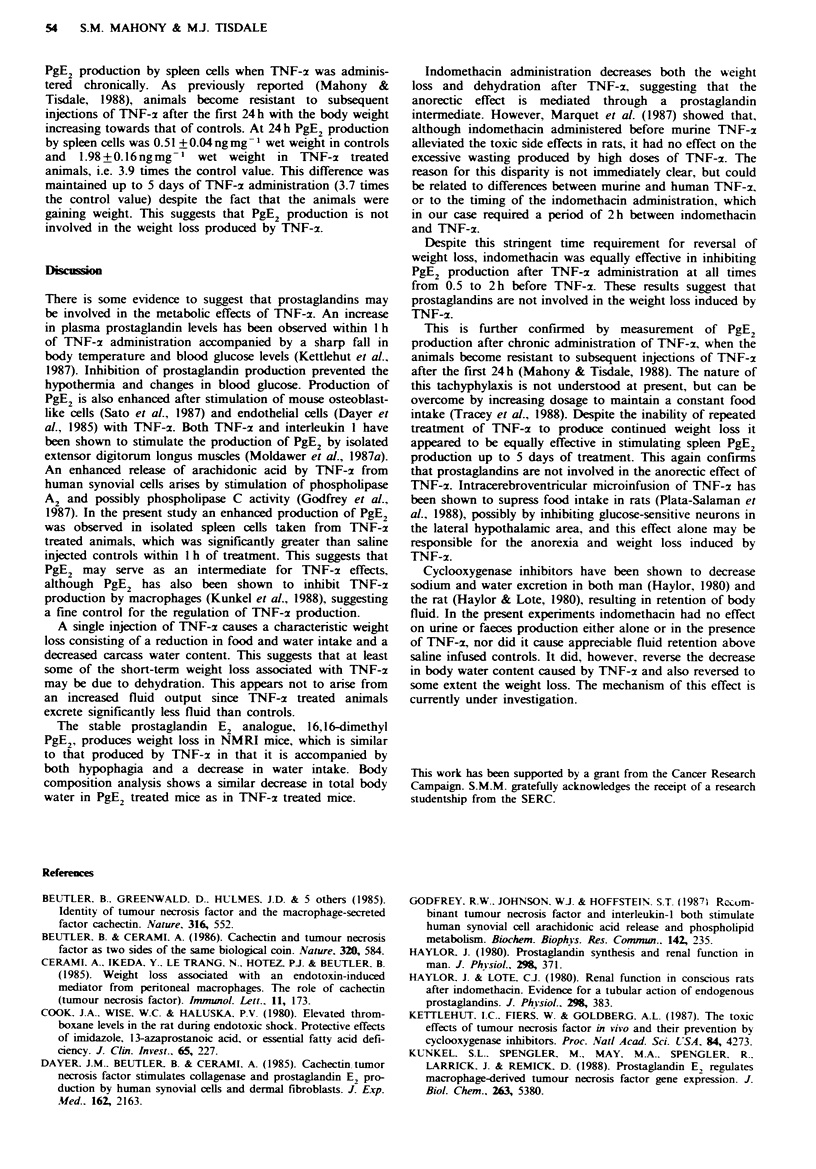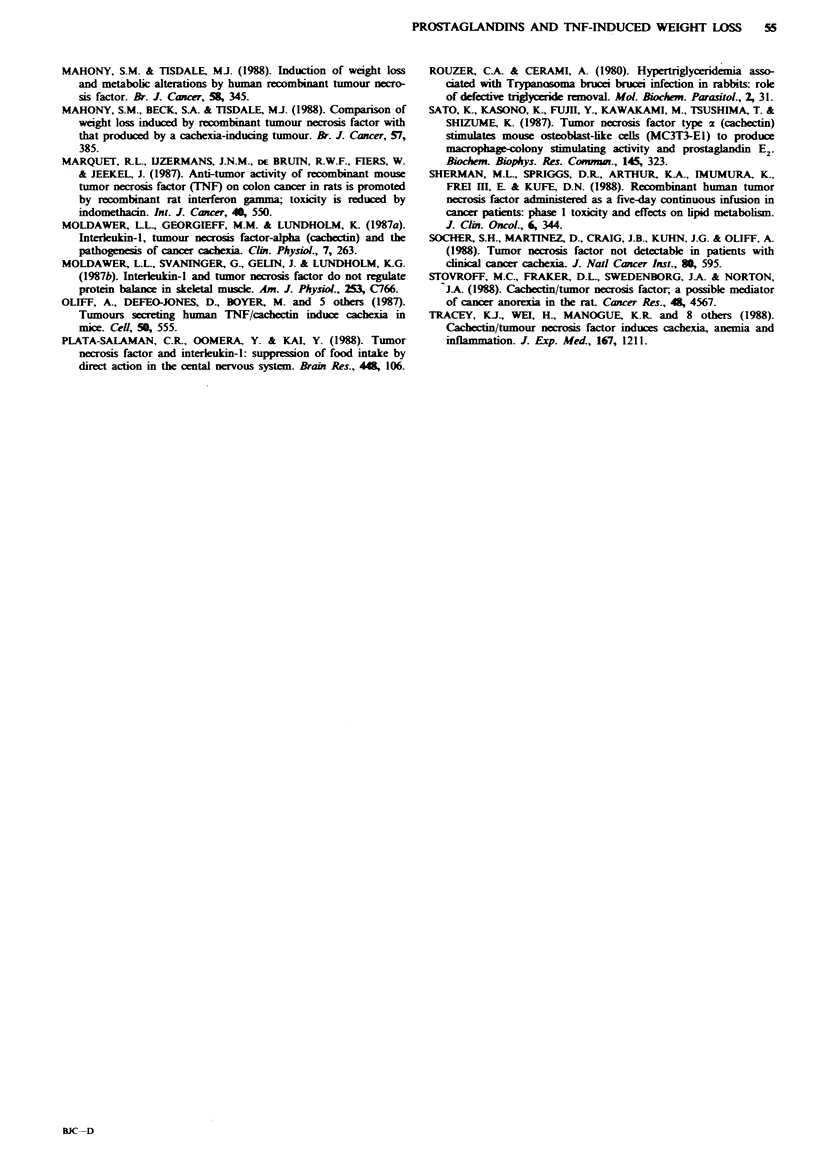# Role of prostaglandins in tumour necrosis factor induced weight loss.

**DOI:** 10.1038/bjc.1989.218

**Published:** 1989-07

**Authors:** S. M. Mahony, M. J. Tisdale

**Affiliations:** CRC Experimental Chemotherapy Group, Aston University, Birmingham, UK.

## Abstract

Administration of either tumour necrosis factor alpha (TNF-alpha) or 16,16-dimethylprostaglandin E2 (PGE2) to female NMRI mice caused a decrease in body weight accompanied by a reduction in both food and water intake and a decrease in carcass water content. A single injection of TNF-alpha caused an enhanced production of PGE2 by spleen cells from treated animals, that was significant within 1 h of treatment, and persisted until at least 6 h. These results suggest that the anorectic effect of TNF-alpha may be mediated by a prostaglandin intermediate. Indomethacin (10 mg kg-1) administered 2 h before TNF-alpha (7.5 x 10(7) U kg-1) caused a significant reduction in the extent of weight loss and inhibited PgE2 production. Administration of indomethacin 0.5-1.5 h before the TNF-alpha had no significant effect on loss of body weight, but still inhibited PgE2 production. Also PgE2 production was still enhanced in response to TNF-alpha administered chronically, despite the inability of prolonged TNF-alpha administration to produce continued loss of body weight. These results suggest that prostaglandins are not involved in the anorectic effect of TNF-alpha.


Br. J. Cancer (1989), 60, 51–55

© The Macmillan Press Ltd., 1989

### Role of prostaglandins in tumour necrosis factor induced weight loss

S.M. Mahony & M.J. Tisdale

*CRC Experimental Chemotherapy Group, Pharmaceutical Sciences Institute, Aston University, Birmingham B4 7ET, UK.*

**Summary** Administration of either tumour necrosis factor alpha (TNF-α) or 16,16-dimethylprostaglandin $E_2$ ($PGE_2$) to female NMRI mice caused a decrease in body weight accompanied by a reduction in both food and water intake and a decrease in carcass water content. A single injection of TNF-α caused an enhanced production of $PGE_2$ by spleen cells from treated animals, that was significant within 1 h of treatment, and persisted until at least 6 h. These results suggest that the anorectic effect of TNF-α may be mediated by a prostaglandin intermediate. Indomethacin ($10\,mg\,kg^{-1}$) administered 2 h before TNF-α ($7.5 \times 10^7\,U\,kg^{-1}$) caused a significant reduction in the extent of weight loss and inhibited $PgE_2$ production. Administration of indomethacin 0.5–1.5 h before the TNF-α had no significant effect on loss of body weight, but still inhibited $PgE_2$ production. Also $PgE_2$ production was still enhanced in response to TNF-α administered chronically, despite the inability of prolonged TNF-α administration to produce continued loss of body weight. These results suggest that prostaglandins are not involved in the anorectic effect of TNF-α.

The catabolic states associated with infection or endotoxemia have been attributed to the production by phagocytic cells of soluble proteins such as interleukin 1 and cachectin (Rouzer & Cerami, 1980; Moldawer *et al.*, 1987*a*; Cerami *et al.*, 1985). A high degree of homology has been shown to exist between the *N*-terminal sequence of mouse cachectin and the *N*-terminal sequence for human tumour necrosis factor-alpha (TNF-α) (Beutler *et al.*, 1985) and the catabolic states have been extended to include also cancer-associated cachexia (Beutler & Cerami, 1986). Severe weight loss and increased mortality have been observed in mice bearing transgenic tumours that persistently secrete human cachectin (Oliff *et al.*, 1987) and chronic administration of sublethal doses of TNF-α to rats caused anorexia, weight loss, depletion of body lipid and protein, a reduction of red blood cell mass, leukocytosis and tissue inflammation (Tracey *et al.*, 1988). However, in a number of studies (Stovroff *et al.*, 1988; Mahony & Tisdale, 1988) administration of TNF-α caused a loss in body weight accompanied by a drop in food and water intake, which was only apparent over the first 24h, after which animals became resistant to subsequent dosing. In addition TNF-α has not been detectable in the serum of patients with clinical cancer cachexia (Socher *et al.*, 1988) and in clinical trials of recombinant human TNF-α there was no clinical evidence of accelerated cachexia, although anorexia was present during administration (Sherman *et al.*, 1988). Also the effects on host metabolism produced by TNF-α appear to differ from that produced by a cachexia-inducing tumour (Mahony *et al.*, 1988).

The toxic and metabolic effects of TNF-α can be blocked by a single injection of the cyclo-oxygenase inhibitors indomethacin or ibuprofen before the TNF-α treatment (Kettlehut *et al.*, 1987). This suggests that some of the effects of TNF-α may be mediated through a prostaglandin intermediate in analogy with septic shock where large increases in circulatory prostaglandins have been reported in a variety of experimental models (Cook *et al.*, 1980). In order to study the role of prostaglandins in the mechanism of weight loss induced by TNF-α we have used NMRI mice, a strain we have utilised to passage a colon adenocarcinoma which induces cachexia in recipient animals (Mahony *et al.*, 1988).

### Materials and methods

### *Animals*

Pure strain female NMRI mice (age 6–8 weeks) were purchased from Banting and Kingman (Hull, UK) and were fed *ad lib* a rat and mouse breeding diet (Pilsbury's Birmingham, UK). All animals were given free access to food and water and both food and water intake were monitored daily.

### *TNF*

Human recombinant TNF-α ($6 \times 10^7\,U\,mg^{-1}$) was kindly donated by Boehringer Ingelheim Ltd (Bracknell, Berks, UK) and was stored at 4°C. The endotoxin content was less than $0.125\,EU\,ml^{-1}$. Fresh solutions of TNF-α were made up daily in 0.9% NaCl and 200 μl of the appropriate concentration ($7.5 \times 10^7\,U\,kg^{-1}$) was injected into the tail veins of female NMRI mice (19–22 g). Controls were injected with 200 μl of 0.9% NaCl. Body weights and food and water intake were monitored at the same time each day.

### *Body water content*

Carcasses were heated at 80°C until a constant weight was achieved. The carcasses were then reweighed and the water content was determined from the difference between the wet and dry weights.

### *Indomethacin administration*

Fresh solutions of indomethacin ($2\,mg\,ml^{-1}$) in arachis oil containing 10% DMSO were made up daily and $10\,mg\,kg^{-1}$ were injected i.p. into female NMRI mice (19–22 g) 0.5–2 h before TNF-α administration ($7.5 \times 10^7\,U\,kg^{-1}$ i.v.). Controls were injected with arachis oil containing 10% DMSO 2 h before 0.9% NaCl administration (200 μl, i.v.). Body weights and food and water intake were monitored over a 24 h period and body composition analysis was performed. Urine and faeces production was measured by placing animals in metabolic cages throughout the experiment.

### *Prostaglandin $E_2$ administration*

Fresh solutions of 16,16-dimethyl $PgE_2$ ($0.125\,mg\,ml^{-1}$) dissolved in triolein were made up daily in 0.9% NaCl and were administered i.p. ($0.5\,mg\,kg^{-1}$) at 6 h intervals (11 a.m., 5 p.m., 11 p.m.) into female NMRI mice ($19 \pm 1$ g). Controls were injected with 200 μl of 0.9% NaCl containing triolein. Body weights and food and water intake were monitored over a 24 h period and body water content was determined. Mice were placed in metabolic cages throughout the experiment and the urine and faeces production was determined.

Received 30 November 1988, and accepted in revised form 13 March 1989.

### $PgE_2$ *analysis*

Male NMRI mice (20–26 g) were injected i.v. with $7.5 \times 10^7$ U kg$^{-1}$ TNF-α or 0.9% NaCl. At specified time points after injection animals were killed by cervical dislocation and the spleens rapidly removed and weighed. Spleens were sliced on filter paper moistened with cold 0.85% NaCl and then placed in a 25 ml flask containing 2 ml of Krebs-Ringer bicarbonate medium containing 1 mg ml$^{-1}$ each of glucose and bovine serum albumin. The slices were first incubated at 37°C for 20 min in a gas phase of 5% $CO_2$/95% $N_2$ and then transferred to flasks containing 5% $CO_2$/95% $O_2$ for a further 15 min. At the end of the incubation spleens were removed and the medium was immediately frozen at −196°C and only defrosted immediately before extraction.

For the determination of $PgE_2$ 1 ml of thawed medium was removed, adjusted to pH 3 to 3.5 with 2 N HCl and extracted twice with 3 ml of ethyl acetate. The extract was evaporated to dryness under a stream of nitrogen and dissolved in 0.025 M phosphate buffer, pH 6.8, containing 0.01 M EDTA, 0.9% NaCl, 0.3% bovine γ-globulin, 0.005% triton × 100 and 0.05% sodium azide and the concentration of $PgE_2$ was determined using a radioimmunoassay (NEN, Dreieich, FR Germany).

### *Statistical analysis*

All results were analysed statistically using the analysis of variance or F ratio.

### Results

We have utilised $PgE_2$ production by spleen cells rapidly removed from TNF-α treated animals as an indirect method of measuring prostaglandin production because of difficulties in measuring plasma levels of $PgE_2$ directly. The radioimmunoassay utilised for these measurements had not previously been tested on mouse plasma and our investigations revealed an inhibition of the binding of $PgE_2$ from mouse plasma with the antibody used in the assay. The results presented in Figure 1 show an enhanced production of $PgE_2$ by spleen cells after administration of TNF-α ($7.5 \times 10^7$ U kg$^{-1}$), which was significantly greater than saline infused controls within 1 h after administration, and remained elevated up to 6 h after treatment. This suggests that some of the metabolic effects of TNF-α may be mediated via prostaglandin production.

**Figure 1** Effect of a single i.v. injection of TNF-α ($7.5 \times 10^7$ U kg$^{-1}$) on $PgE_2$ production by spleen cells. Spleens were rapidly removed from control (□) and TNF-α (◆) treated animals and $PgE_2$ production *in vitro* was determined by a radioimmunoassay. The values represent means ± s.e.m. for 5–6 animals. *$P \leqslant 0.001$ from controls.

**Figure 2** Effect of TNF-α and 16,16-dimethyl $PgE_2$ administration on body weight change (**a**), food (▩) and water (▨) consumption (**b**) and body water content (**c**). Female NMRI mice (19.0 ± 1.0 g) were given either a single i.v. injection of TNF-α ($7.5 \times 10^7$ U kg$^{-1}$) or dimethyl $PgE_2$ (0.5 mg kg$^{-1}$) administered i.p. at three 6-hourly intervals and the parameters were measured as described in methods. The values represent means ± s.e.m. for nine animals. *$P \leqslant 0.001$ from controls.

Administration of TNF-α ($7.5 \times 10^7$ U kg$^{-1}$) produced a decrease in body weight within the first 24 h after administration (Figure 2a), accompanied by a decrease in food and water intake (Figure 2b). Body composition analysis revealed a decrease in the body water content of TNF-α treated mice, when compared with saline infused controls (Figure 2c). Essentially similar results were obtained after administration of the stable $PgE_2$ analogue 16,16-dimethyl $PgE_2$ at a dose of 0.5 mg kg$^{-1}$ administered i.p. three times daily. No effect was seen after a dose of 1.25 mg kg$^{-1}$ administered as a single i.v. injection. Thus after 24 h $PgE_2$ treated animals showed a reduction in body weight (Figure 2a) which was similar to that produced by TNF-α in that it was accompanied by a reduction in both food and water intake (Figure 2b) and a decrease in the body water content (Figure

**Table I** Effect of TNF-α and indomethacin alone and in combination on body weight, food and water intake and $PgE_2$ production by spleen cells

| Treatment | *Body weight change (g)* | *Food intake (g)* | *Water intake (ml)* | *$PgE_2$ (ng mg$^{-1}$ wet weight)* |
|---|---|---|---|---|
| Controls, i.v. saline | 0.00±0.17 | 4.39±0.26 | 7.04±0.46 | 0.69±0.11 |
| i.v. saline + 10 mg kg$^{-1}$ indomethacin | +0.19±0.13[d] | 4.89±0.22 | 5.77±0.36 | 0.17±0.41 |
| TNF-α ($7.5\times10^7$ U kg$^{-1}$) | −1.35±0.14[a] | 2.30±0.16[a] | 3.40±0.33[a] | 1.60±0.31[a] |
| TNF-α ($7.5\times10^7$ U kg$^{-1}$) + 10 mg kg$^{-1}$ indomethacin 30 min before TNF-α | −1.63±0.19 | 1.63±0.49 | 4.5±0.33 | 0.44±0.17[c] |
| TNF-α ($7.5\times10^7$ U kg$^{-1}$) + 10 mg kg$^{-1}$ indomethacin 1.5 h before TNF-α | −1.23±0.54 | 1.98±0.46 | 5.75±0.36 | 0.53±0.47[c] |
| TNF-α ($7.5\times10^7$ U kg$^{-1}$) + 10 mg kg$^{-1}$ indomethacin 2 h before TNF-α | −1.53±0.11[b,c] | 2.76±0.17[e,f] | 3.91±0.41[f] | 0.43±0.27[c] |

Results represent means ± s.e.m. for 5–16 animals for each group.

[a]$P\leqslant0.001$ from controls; [b]$P\leqslant0.005$ from controls; [c]$P\leqslant0.001$ from TNF-α alone; [d]$P\leqslant0.001$ from TNF-α/indomethacin 2 h; [e]$P\leqslant0.05$ from TNF-α; [f]$P\leqslant0.001$ from indomethacin 2 h.

2c). These results suggest that the effect of TNF-α on body weight may be mediated via a prostaglandin intermediate, and that it may be possible to reverse the effects by the inhibition of prostaglandin synthesis.

Administration of indomethacin (10 mg kg$^{-1}$) 2 h before a single injection of TNF-α ($7.5\times10^7$ U kg$^{-1}$) caused a significant reduction in the TNF-α induced weight loss (Table I). The time of administration of indomethacin appeared to be critical since no weight reversal was observed 0.5 or 1.5 h before the TNF-α (Table I). When compared with indomethacin treated controls the decrease in water intake in the 2 h indomethacin/TNF-α treated mice (32%) was not as great as in the TNF-α treated mice compared with saline infused controls (52%), although the food intake was reduced to about the same extent in both cases (44% and 48% respectively, Table I) compared with the respective controls. There was a significant increase in food consumption of the TNF-α/indomethacin treated mice when compared with TNF-α treatment alone, and body composition analysis showed an increase in the total body water content of the TNF-α/indomethacin group when compared with indomethacin alone (Figure 3). This difference was not explained by decreased excretion of urine (Table II). Animals treated with TNF-α had a significant reduction in the excretion of both urine and faeces, indicating that the decrease in body water content did not arise from a diuretic effect of TNF-α. 16,16-Dimethyl $PgE_2$ also caused a reduction in urine and faeces production (Table II). Indomethacin had no effect on urine or faeces production in control animals, although there was a small increase in faeces production in TNF-α treated animals.

**Figure 3** Effect of indomethacin on the body water content of TNF-α treated mice. Animals were given either 0.9% NaCl i.v., $7.5\times10^7$ U kg$^{-1}$ TNF-α, i.v., 10 mg kg$^{-1}$ indomethacin i.p. 2 h before 0.9% NaCl i.v., or 10 mg kg$^{-1}$ indomethacin i.p. 2 h before $7.5\times10^7$ U kg$^{-1}$ TNF-α i.v. The values represent mean ± s.e.m. for seven animals. *$P\leqslant0.001$ from TNF-α treated animals, †$P\leqslant0.001$ from saline injected controls.

The effect of indomethacin on $PgE_2$ production by spleen cells from TNF-α treated animals is shown in Table I. All values were measured 2 h after TNF-α administration since $PgE_2$ production in response to TNF-α was significantly elevated at this point (Figure 1). Indomethacin inhibited $PgE_2$ production in response to $PgE_2$ irrespective of the time of administration with respect to TNF-α, although the reversal of body weight loss was highly dependent on the time of administration (Table I). This suggests that indomethacin did not reverse the TNF-α induced weight loss as a result of inhibition of prostaglandin synthesis and that prostaglandin production was not necessary for weight loss to occur.

This conclusion was also substantiated by measurement of

**Table II** Effect of TNF-α, indomethacin and 16,16-dimethyl $PgE_2$ on excretion of urine and faeces

| Treatment | *Urine volume (ml)* | *Wet faeces weight (g)* | *Dry faeces weight (g)* | *Total fluid excretion (ml)* |
|---|---|---|---|---|
| Controls, i.v. saline | 1.28±0.3 | 2.13±0.9 | 1.15±0.4 | 2.05±0.34 |
| 10 mg kg$^{-1}$ indomethacin | 0.97±0.2 | 2.24±0.3 | 1.18±0.2 | 2.00±0.30 |
| TNF-α ($7.5\times10^7$ U kg$^{-1}$) | 0.44±0.2[b] | 0.50±0.08[a] | 0.32±0.06[b] | 0.62±0.16[c] |
| TNF-α ($7.5\times10^7$ U kg$^{-1}$) + 10 mg kg$^{-1}$ indomethacin | 0.66±0.2 | 0.80±0.13[d,e] | 0.51±0.08[d,e] | 0.95±0.26[d] |
| Dimethyl $PgE_2$, 0.5 mg kg$^{-1}$ 3 × daily | 0.75±0.2 | 0.97±0.2[a] | 0.34±0.11[b] | 1.38±0.12[a] |

Results are expressed as means ± s.e.m. for 6–7 animals per group.
[a]$P\leqslant0.05$ from saline controls; [b]$P\leqslant0.005$ from saline controls; [c]$P\leqslant0.001$ from saline controls; [d]$P\leqslant0.001$ from indomethacin controls; [e]$P\leqslant0.005$ from TNF-α alone.

$PgE_2$ production by spleen cells when TNF-α was administered chronically. As previously reported (Mahony & Tisdale, 1988), animals become resistant to subsequent injections of TNF-α after the first 24 h with the body weight increasing towards that of controls. At 24 h $PgE_2$ production by spleen cells was $0.51 \pm 0.04\,ng\,mg^{-1}$ wet weight in controls and $1.98 \pm 0.16\,ng\,mg^{-1}$ wet weight in TNF-α treated animals, i.e. 3.9 times the control value. This difference was maintained up to 5 days of TNF-α administration (3.7 times the control value) despite the fact that the animals were gaining weight. This suggests that $PgE_2$ production is not involved in the weight loss produced by TNF-α.

### Discussion

There is some evidence to suggest that prostaglandins may be involved in the metabolic effects of TNF-α. An increase in plasma prostaglandin levels has been observed within 1 h of TNF-α administration accompanied by a sharp fall in body temperature and blood glucose levels (Kettlehut *et al.*, 1987). Inhibition of prostaglandin production prevented the hypothermia and changes in blood glucose. Production of $PgE_2$ is also enhanced after stimulation of mouse osteoblast-like cells (Sato *et al.*, 1987) and endothelial cells (Dayer *et al.*, 1985) with TNF-α. Both TNF-α and interleukin 1 have been shown to stimulate the production of $PgE_2$ by isolated extensor digitorum longus muscles (Moldawer *et al.*, 1987*a*). An enhanced release of arachidonic acid by TNF-α from human synovial cells arises by stimulation of phospholipase $A_2$ and possibly phospholipase C activity (Godfrey *et al.*, 1987). In the present study an enhanced production of $PgE_2$ was observed in isolated spleen cells taken from TNF-α treated animals, which was significantly greater than saline injected controls within 1 h of treatment. This suggests that $PgE_2$ may serve as an intermediate for TNF-α effects, although $PgE_2$ has also been shown to inhibit TNF-α production by macrophages (Kunkel *et al.*, 1988), suggesting a fine control for the regulation of TNF-α production.

A single injection of TNF-α causes a characteristic weight loss consisting of a reduction in food and water intake and a decreased carcass water content. This suggests that at least some of the short-term weight loss associated with TNF-α may be due to dehydration. This appears not to arise from an increased fluid output since TNF-α treated animals excrete significantly less fluid than controls.

The stable prostaglandin $E_2$ analogue, 16,16-dimethyl $PgE_2$, produces weight loss in NMRI mice, which is similar to that produced by TNF-α in that it is accompanied by both hypophagia and a decrease in water intake. Body composition analysis shows a similar decrease in total body water in $PgE_2$ treated mice as in TNF-α treated mice.

Indomethacin administration decreases both the weight loss and dehydration after TNF-α, suggesting that the anorectic effect is mediated through a prostaglandin intermediate. However, Marquet *et al.* (1987) showed that, although indomethacin administered before murine TNF-α alleviated the toxic side effects in rats, it had no effect on the excessive wasting produced by high doses of TNF-α. The reason for this disparity is not immediately clear, but could be related to differences between murine and human TNF-α, or to the timing of the indomethacin administration, which in our case required a period of 2 h between indomethacin and TNF-α.

Despite this stringent time requirement for reversal of weight loss, indomethacin was equally effective in inhibiting $PgE_2$ production after TNF-α administration at all times from 0.5 to 2 h before TNF-α. These results suggest that prostaglandins are not involved in the weight loss induced by TNF-α.

This is further confirmed by measurement of $PgE_2$ production after chronic administration of TNF-α, when the animals become resistant to subsequent injections of TNF-α after the first 24 h (Mahony & Tisdale, 1988). The nature of this tachyphylaxis is not understood at present, but can be overcome by increasing dosage to maintain a constant food intake (Tracey *et al.*, 1988). Despite the inability of repeated treatment of TNF-α to produce continued weight loss it appeared to be equally effective in stimulating spleen $PgE_2$ production up to 5 days of treatment. This again confirms that prostaglandins are not involved in the anorectic effect of TNF-α. Intracerebroventricular microinfusion of TNF-α has been shown to supress food intake in rats (Plata-Salaman *et al.*, 1988), possibly by inhibiting glucose-sensitive neurons in the lateral hypothalamic area, and this effect alone may be responsible for the anorexia and weight loss induced by TNF-α.

Cyclooxygenase inhibitors have been shown to decrease sodium and water excretion in both man (Haylor, 1980) and the rat (Haylor & Lote, 1980), resulting in retention of body fluid. In the present experiments indomethacin had no effect on urine or faeces production either alone or in the presence of TNF-α, nor did it cause appreciable fluid retention above saline infused controls. It did, however, reverse the decrease in body water content caused by TNF-α and also reversed to some extent the weight loss. The mechanism of this effect is currently under investigation.

This work has been supported by a grant from the Cancer Research Campaign. S.M.M. gratefully acknowledges the receipt of a research studentship from the SERC.

### References

BEUTLER, B., GREENWALD, D., HULMES, J.D. & 5 others (1985). Identity of tumour necrosis factor and the macrophage-secreted factor cachectin. *Nature*, **316**, 552.

BEUTLER, B. & CERAMI, A. (1986). Cachectin and tumour necrosis factor as two sides of the same biological coin. *Nature*, **320**, 584.

CERAMI, A., IKEDA, Y., LE TRANG, N., HOTEZ, P.J. & BEUTLER, B. (1985). Weight loss associated with an endotoxin-induced mediator from peritoneal macrophages. The role of cachectin (tumour necrosis factor). *Immunol. Lett.*, **11**, 173.

COOK, J.A., WISE, W.C. & HALUSKA, P.V. (1980). Elevated thromboxane levels in the rat during endotoxic shock. Protective effects of imidazole, 13-azaprostanoic acid, or essential fatty acid deficiency. *J. Clin. Invest.*, **65**, 227.

DAYER, J.M., BEUTLER, B. & CERAMI, A. (1985). Cachectin/tumor necrosis factor stimulates collagenase and prostaglandin $E_2$ production by human synovial cells and dermal fibroblasts. *J. Exp. Med.*, **162**, 2163.

GODFREY, R.W., JOHNSON, W.J. & HOFFSTEIN, S.T. (1987). Recombinant tumour necrosis factor and interleukin-1 both stimulate human synovial cell arachidonic acid release and phospholipid metabolism. *Biochem. Biophys. Res. Commun.*, **142**, 235.

HAYLOR, J. (1980). Prostaglandin synthesis and renal function in man. *J. Physiol.*, **298**, 371.

HAYLOR, J. & LOTE, C.J. (1980). Renal function in conscious rats after indomethacin. Evidence for a tubular action of endogenous prostaglandins. *J. Physiol.*, **298**, 383.

KETTLEHUT, I.C., FIERS, W. & GOLDBERG, A.L. (1987). The toxic effects of tumour necrosis factor *in vivo* and their prevention by cyclooxygenase inhibitors. *Proc. Natl Acad. Sci. USA*, **84**, 4273.

KUNKEL, S.L., SPENGLER, M., MAY, M.A., SPENGLER, R., LARRICK, J. & REMICK, D. (1988). Prostaglandin $E_2$ regulates macrophage-derived tumour necrosis factor gene expression. *J. Biol. Chem.*, **263**, 5380.

MAHONY, S.M. & TISDALE, M.J. (1988). Induction of weight loss and metabolic alterations by human recombinant tumour necrosis factor. *Br. J. Cancer*, **58**, 345.

MAHONY, S.M., BECK, S.A. & TISDALE, M.J. (1988). Comparison of weight loss induced by recombinant tumour necrosis factor with that produced by a cachexia-inducing tumour. *Br. J. Cancer*, **57**, 385.

MARQUET, R.L., IJZERMANS, J.N.M., DE BRUIN, R.W.F., FIERS, W. & JEEKEL, J. (1987). Anti-tumor activity of recombinant mouse tumor necrosis factor (TNF) on colon cancer in rats is promoted by recombinant rat interferon gamma; toxicity is reduced by indomethacin. *Int. J. Cancer*, **40**, 550.

MOLDAWER, L.L., GEORGIEFF, M.M. & LUNDHOLM, K. (1987*a*). Interleukin-1, tumour necrosis factor-alpha (cachectin) and the pathogenesis of cancer cachexia. *Clin. Physiol.*, **7**, 263.

MOLDAWER, L.L., SVANINGER, G., GELIN, J. & LUNDHOLM, K.G. (1987*b*). Interleukin-1 and tumor necrosis factor do not regulate protein balance in skeletal muscle. *Am. J. Physiol.*, **253**, C766.

OLIFF, A., DEFEO-JONES, D., BOYER, M. and 5 others (1987). Tumours secreting human TNF/cachectin induce cachexia in mice. *Cell*, **50**, 555.

PLATA-SALAMAN, C.R., OOMERA, Y. & KAI, Y. (1988). Tumor necrosis factor and interleukin-1: suppression of food intake by direct action in the cental nervous system. *Brain Res.*, **448**, 106.

ROUZER, C.A. & CERAMI, A. (1980). Hypertriglyceridemia associated with Trypanosoma brucei brucei infection in rabbits: role of defective triglyceride removal. *Mol. Biochem. Parasitol.*, **2**, 31.

SATO, K., KASONO, K., FUJII, Y., KAWAKAMI, M., TSUSHIMA, T. & SHIZUME, K. (1987). Tumor necrosis factor type α (cachectin) stimulates mouse osteoblast-like cells (MC3T3-E1) to produce macrophage-colony stimulating activity and prostaglandin $E_2$. *Biochem. Biophys. Res. Commun.*, **145**, 323.

SHERMAN, M.L., SPRIGGS, D.R., ARTHUR, K.A., IMUMURA, K., FREI III, E. & KUFE, D.N. (1988). Recombinant human tumor necrosis factor administered as a five-day continuous infusion in cancer patients: phase 1 toxicity and effects on lipid metabolism. *J. Clin. Oncol.*, **6**, 344.

SOCHER, S.H., MARTINEZ, D., CRAIG, J.B., KUHN, J.G. & OLIFF, A. (1988). Tumor necrosis factor not detectable in patients with clinical cancer cachexia. *J. Natl Cancer Inst.*, **80**, 595.

STOVROFF, M.C., FRAKER, D.L., SWEDENBORG, J.A. & NORTON, J.A. (1988). Cachectin/tumor necrosis factor; a possible mediator of cancer anorexia in the rat. *Cancer Res.*, **48**, 4567.

TRACEY, K.J., WEI, H., MANOGUE, K.R. and 8 others (1988). Cachectin/tumour necrosis factor induces cachexia, anemia and inflammation. *J. Exp. Med.*, **167**, 1211.